# Transcription Factor σ^B^ Plays an Important Role in the Production of Extracellular Membrane-Derived Vesicles in *Listeria monocytogenes*

**DOI:** 10.1371/journal.pone.0073196

**Published:** 2013-08-20

**Authors:** Jung Hwa Lee, Chi-Won Choi, Taewon Lee, Seung Il Kim, Je-Chul Lee, Ji-Hyun Shin

**Affiliations:** 1 Department of Microbiology, School of Medicine, Kyungpook National University, Daegu, Republic of Korea; 2 Division of Life Science, Korea Basic Science Institute, Daejeon, Republic of Korea; 3 Department of Information and Mathematics, Korea University, Sejong, Republic of Korea; 4 Department of Pharmacology, College of Medicine, Dankook University, Cheonan, Republic of Korea; University Medical Center Utrecht, Netherlands

## Abstract

Gram-negative bacteria produce extracellular outer membrane vesicles (OMVs) that interact with host cells. Unlike Gram-negative bacteria, less is known about the production and role of extracellular membrane vesicles (MVs) in Gram-positive bacteria. The food-borne pathogen *Listeria monocytogenes* can survive under extreme environmental and energy stress conditions and the transcription factor σ^B^ is involved in this survival ability. Here, we first determined the production of MVs from *L. monocytogenes* and evaluated whether general stress transcription factor σ^B^ affected production of MVs in *L. monocytogenes. L. monocytogenes* secreted MVs during *in vitro* broth culture. The wild-type strain actively produced MVs approximately nine times more and also produced more intact shapes of MVs than those of the isogenic Δ*sigB* mutant. A proteomic analysis showed that 130 and 89 MV proteins were identified in the wild-type and Δ*sigB* mutant strains, respectively. Wild-type strain-derived MVs contained proteins regulated by σ^B^ such as transporters (OpuCA and OpuCC), stress response (Kat), metabolism (LacD), translation (InfC), and cell division protein (FtsZ). Gene Ontology (GO) enrichment analysis showed that wild-type-derived MV proteins corresponded to several GO terms, including response to stress (heat, acid, and bile resistance) and extracellular polysaccharide biosynthetic process, but not the Δ*sigB* mutant. Internalin B (InlB) was almost three times more contained in MVs derived from the wild-type strain than in MVs derived from the Δ*sigB* mutant. Taken together, these results suggest that σ^B^ plays a pivotal role in the production of MVs and protein profiles contained in MVs. *L. monocytogenes* MVs may contribute to host infection and survival ability under various stressful conditions.

## Introduction

*Listeria monocytogenes* is a Gram-positive, facultative intracellular bacterium that causes listeriosis. This organism is well-known for its robust survival under various environmental and energy stress conditions such as acid stress [1], osmotic stress [2,3], and carbon starvation [4]. The general stress transcription factor σ^B^ largely contributes to resistance properties to these stresses. σ^B^ is important for survival of *L. monocytogenes* during food processing and also plays an important role in host infection, including survival in the gastrointestinal tract with low acidic and high osmotic pressure, and invasion of intestinal epithelium. Example proteins include GadB, a product that controls expression of glutamate decarboxylase acid stress resistance; OpuCA, similar to the glycine betaine-carnitine-choline ABC transporter for osmotic stress resistance; Bsh, which contributes to bile salt resistance; Internalin A (InlA) and Internalin B (InlB), which are required for invasion into intestinal lumen cells; and PrfA, a master virulence regulator [5–8]. Thus, the σ^B^ null mutant shows reduced resistance to acid, salt, antibiotics, temperature, and carbon starvation stresses [1,2,7,9], and it shows decreased virulence in guinea pigs infected *via* the gastrointestinal route [10].

A wide variety of Gram-negative bacterial species produce and release spherical and bilayered nanovesicles into the surrounding environment, called outer membrane vesicles (OMVs). As a bacterial secretion system, OMVs contribute to cell-free intercellular communication, detoxification of environmental stresses, killing of competitors, and transfer of bacterial effectors between bacteria or into host cells [11,12]. As an example, *Pseudomonas* *aerugonosa* OMVs contain various virulence factors, such as peptidoglycan hydrolase, phospholipase C, hemolysin, alkaline phosphatase, and antibacterial factors [13], including murin hydrolase [14]. Moreover, pathogen-derived OMVs contain various toxins, including cytolysin A from enterohemorrhagic Escherichia coli and *Salmonella* *typhi* [15], vacuolating cytotoxin from *Helicobacter pylori* [16], and Shiga toxin from *Shigella dysenteriae* [17].

Gram-positive bacteria also produce and secrete membrane-derived vesicles (MVs), but the pathophysiological function of MVs has not been elucidated. According to recent reports, *Staphylococcus aureus* [18,19], *Bacillus* spp. [20,21], and *Mycobacterium* *ulcerans* [22] release MVs. MVs from *B. anthracis* contain biologically active toxins, such as anthrolysin [21] and *S. aureus*-derived MV components that are delivered to host cells and induce cytotoxicity in host cells [19].

In this study, we determined whether *L. monocytogenes* produced MVs during in vitro broth culture. Next, MVs derived from wild-type *L. monocytogenes* and its isogenic Δ*sigB* mutant were subjected to proteomic analysis to investigate the role of σ^B^ in the production of MVs and in the MV proteins profiles. Our results demonstrate that *L. monocytogenes* produces MVs and that σ^B^ plays a pivotal role in the production of MVs and in the *L. monocytogenes* MV protein profiles.

## Materials and Methods

### Bacterial strains and β-galactosidase accumulation assay

Two *L. monocytogenes* strains, wild-type strain 10403S (serotype 1/2a) and an isogenic Δ*sigB* mutant, were used in this study. These strains were obtained from Martin Wiedmann (Cornell University). *L. monocytogenes* cells were maintained on brain-heart infusion (BHI) (BD Science, Franklin Lakes, NJ, USA) agar or broth, and were grown at 37°C. σ^B^ activity was measured in wild type and Δ*sigB* mutant *L. monocytogenes* carrying the reporter gene fusion (σ^B^-dependent *opuCA* promoter and a *lacZ* reporter gene) during the bacterial growth by measuring the specific activity of β-galactosidase. These strains were constructed in our previous study [9]. β-galactosidase assays were performed as described by Miller [23]. Briefly, samples were collected at the indicated times by centrifugation for 1 min at 6,000 *g*. Cells were washed with Z buffer [23] and permeablized by vigorous voltexing for 30 s using sodium dodecyl sulfate and chloroform, then incubated at 28^o^C with the o-nitrophenyl β-D-galactopyranoside substrate. Reactions were stopped by the addition of 0.5 ml of 1M Na _2_CO_3_, and the mixes were centrifuged to remove cellular interference before reading absorbance at 420 nm. Protein levels were determined using the Bio-Rad Protein Assay reagent (Bio-Rad, USA). Specific activity was defined as ΔA_420 nm_ × 1,000 min^-1^ mg^-1^ of protein.

### Isolation of MVs from culture supernatants

The extracellular MVs produced by *L. monocytogenes* were prepared from bacterial culture supernatants as described previously [15,24]. Two bacterial strains, the wild type and Δ*sigB* mutant, were inoculated into 500 ml of BHI broth and grown until the optical density at 600 nm (OD600) reached 2.0 at 37°C with shaking. After the bacterial cells were removed by centrifugation at 6,000 *g* for 15 min, the supernatants were filtered through a QuixStand Benchtop System (GE Healthcare, Piscataway, NJ, USA) using a 0.2 µm hollow fiber membrane (GE Healthcare) to remove bacterial debris, and the samples were then concentrated by ultrafiltration with a QuixStand Benchtop System using a 500 kDa hollow fiber membrane (GE Healthcare) to exclude molecules with a molecular mass < 500 kDa. The MV fractions were ultracentrifuged at 150,000 *g* for 3 h at 4°C, and the pellets containing the MVs were resuspended in phosphate-buffered saline (PBS). The protein concentration was determined using a modified BCA assay (Thermo Scientific, Rockford, IL, USA). The purified MVs were checked for sterility and stored at -80°C until use. Three independent experiments were conducted to determine the extracellular MV production from culture supernatants of the wild type and Δ*sigB* mutant.

### Transmission electronic microscopy (TEM) analysis

The purified MV samples were applied to copper grids (Electron Microscopy Sciences, Hatfield, PA, USA) and stained with 2% uranyl acetate. The samples were then visualized by TEM (Hitchi H-7500, Hitachi, Tokyo, Japan) operated at 120 kV.

### Proteomic analysis of MVs produced by L. monocytogenes

Protein samples were separated by 12% sodium dodecyl sulfate polyacrylamide gel electrophoresis (SDS-PAGE) (mini-PROTEAN system, Bio-Rad, Hercules, CA, USA). A 10 µg protein sample was applied to each lane, and the gels were stained with Coomassie Brilliant Blue R-250 (Bio-Rad). In-gel digestion was conducted in accordance with a method described previously [25]. Gels were fractionated into six parts according to molecular weight. Each part was digested with trypsin (0.1 µg) for 16 h at 37°C after reduction and alkylation of the cysteines of the proteins. Digested peptides were extracted with an extraction solution (50 mM ammonium bicarbonate, 50% acetonitrile, and 5% trifluoroacetic acid). Digested peptides were resolved in 10 µl of sample solution containing 0.02% formic acid and 0.5% acetic acid. The peptide samples (5 µl) were concentrated on a Easy-column (L 2 cm, ID 100 µm, 120 Å, C18-A1) trapping column (PROXEON, Odense, Denmark). Peptides were eluted from the column and directed onto a Easy-column (L 10 cm, ID 75 µm, 120 Å, C18-A2) reverse phase column (PROXEON) at a flow rate of 200 nl/min. Peptides were eluted in a gradient of 0–65% acetonitrile for 120 min. All MS and MS/MS spectra in the LTQ-Velos ESI ion trap mass spectrometer (Thermo Scientific) were acquired in a data-dependent mode. Each full MS (m/z range of 300 to 2,000) scan was followed by three MS/MS scans of the most abundant precursor ions in the MS spectrum with dynamic exclusion enabled. MS/MS spectra were searched with MASCOT to identify the proteins (Matrix Science, www.matrixscience.com). The genome sequence of *L. monocytogenes* from NCBI (http://www.ncbi.nlm.nih.gov/) and the decoy sequence database were used as the database for protein identification. The mass tolerance of parent or fragment ions was 0.8 Da. Cabamidomethylation of cysteine and oxidation of methionine were considered in the MS/MS analysis as variable modifications of tryptic peptides.

### Gene ontology (GO) enrichment analysis

GO enrichment analysis was performed using the David service (http://david.abcc.ncifcrf.gov/) to identify the biological functions of the identified MVs proteins derived from wild type and Δ*sigB* mutant *L. monocytogenes* [26]. The GO terms enrichment analysis of the identified MV proteins with UniProt accessions number was performed in terms of molecular functions (MF), biological processes (BP) and cellular components (CC). *P*-values to measure gene enrichment in annotation terms were calculated using a modified Fisher’s exact test [27,28]. *P*-values < 0.05 were considered significant.

### SDS-PAGE and Western blot analysis

Both wild-type and Δ*sigB* mutant cells were cultured in BHI broth at 37°C with shaking. The cells (OD_600_ = 2) were pelleted by centrifugation at 6,000 *g* for 10 min and washed twice with PBS. The cell pellet and purified MVs were resuspended in SDS-PAGE sample buffer (1 M Tris HCl pH 6.8, 10% SDS, 1% bromophenol blue, glycerol, and β-mecaptoethanol) and boiled for 10 min. The samples were separated on 10% SDS-PAGE, followed by electrotransfer onto nitrocellulose membranes (Hybond-ECL, Amersham Pharmacia Biotech, Parsippany, NJ, USA). The blots were blocked in 5% non-fat skim milk and incubated with a rabbit anti-listeriolysin O (LLO) antibody (Abcam, Cambridge, MA, USA) and mouse anti-InlB antibody, which were produced by Cosmogene Tech (Seoul, Korea). LLO and InlB proteins were visualized by incubation with horseradish peroxidase-conjugated goat anti-rabbit and anti-mouse IgG antibodies, respectively (Santa Cruz Biotechnology, Santa Cruz, CA, USA), followed by enhanced chemiluminescence (ECL plus; Amersham Pharmacia Biotech) according to the manufacturer’s instructions. The band intensities of the immunoblotted products were measured using ImageJ software (NIH, Bethesda, MD, USA).

## Results

### MV production in the wild-type and ∆sigB mutant L. monocytogenes

**Figure 1:**
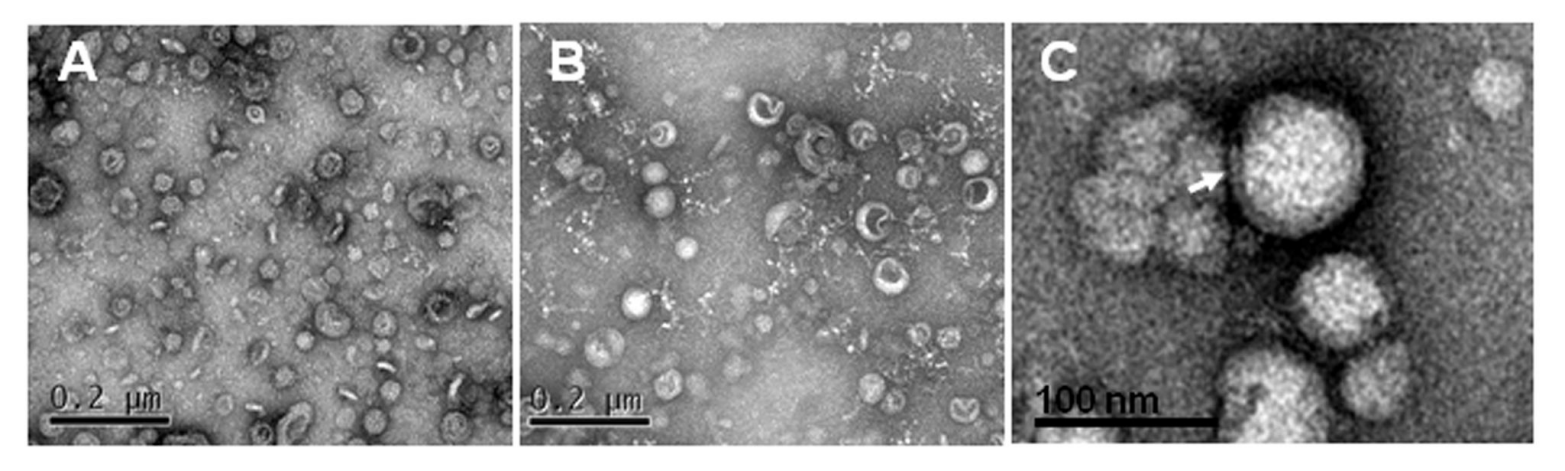
Extracellular membrane vesicles (MVs) produced by *L. monocytogenes*. Transmission electron micrograph of MVs prepared from wild-type (A) and the isogenic ∆*sigB* mutant of *L. monocytogenes* (B) cultured in BHI broth. (C) Arrow indicates bilayered structure.

To evaluate if *L. monocytogenes* produces extracellular MVs, both the wild-type *L. monocytogenes* and its isogenic ∆*sigB* mutant were cultured in BHI broth and MVs were harvested from the each culture supernatant. Both *L. monocytogenes* strains produced MVs, but the wild-type strain actively produced MVs approximately nine-times more than that of the ∆*sigB* mutant (121±6.2 µg/l vs. 14±0.4 µg/l) (Figure 1A and 1B). Moreover, we measured σ^B^ activity in the cells during the growth until an OD600 of 2. The specific activity of β-galactosidase was rapidly induced after entering the stationary phase and then showed a constant level in the wild type strain. However, the specific activity of β-galactosidase was not observed in the Δ*sigB* mutant *L. monocytogenes* (Figure S1). We observed the shapes and sizes of MVs by TEM. The wild-type strain produced intact shapes of MVs as compared to those of the ∆*sigB* mutant, which produced partially wrinkled shaped MVs (Figure 1A and 1B). MVs from *L. monocytogenes* had double membrane spheres ranging from 20 to 100 nm in diameter (Figure 1A–C).

### Protein profiles in the MVs derived from wild-type and ∆sigB mutant L. monocytogenes

**Figure 2:**
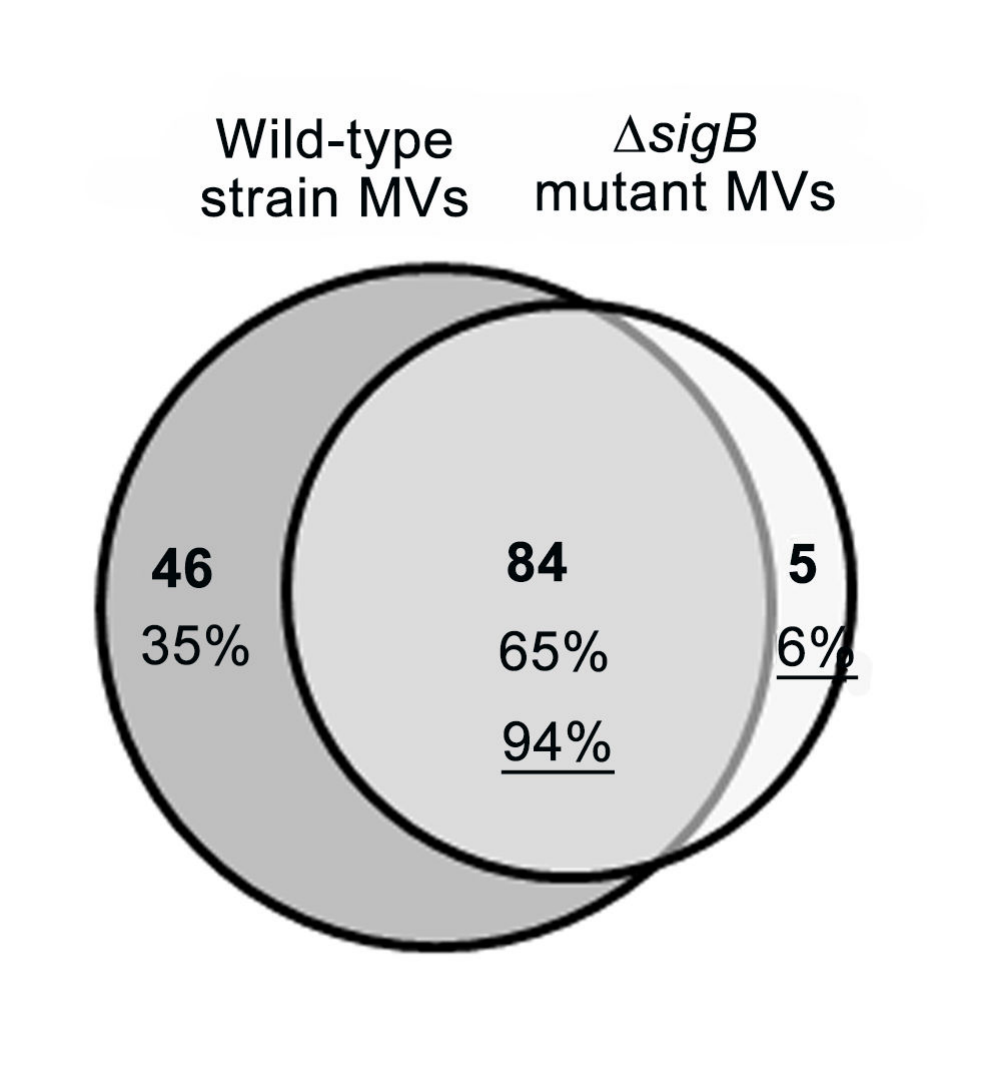
Venn diagram of extracellular membrane vesicle (MV) proteins identified by LC-ESI-MS/MS. Of the 130 proteins, 46 (35%) were identified only in wild-type *L. monocytogenes* MVs and of the 89 proteins, five (6%) were identified only in the Δ*sigB* mutant MVs. Eighty-four MV proteins were identified commonly in the wild-type and Δ*sigB* mutant of *L. monocytogenes*.

Purified MVs were analyzed by LC-ESI-MS/MS to identify proteins contained in the MVs. Three independent analyses were performed for the MVs derived from wild-type and ∆*sigB* mutant *L. monocytogenes*. Proteins only appearing in all three analyses were considered identified proteins for each strain. The analysis identified 130 proteins from the MVs of wild-type strain and 89 from the MVs of ∆*sigB* mutant *L. monocytogenes* (Figure 2). Among the proteins identified in the MVs, 84 were commonly identified in both strains (Table S1). Forty-six and five unique proteins were identified in the MVs of wild type and ∆*sigB* mutant *L. monocytogenes*, respectively (Tables S2 and S3). Of the 46 proteins derived from the wild-type strain, 18 are known as σ^B^-dependent proteins in *L. monocytogenes* (Table 1) [5,6,29]. Overall, these identified MV proteins were transporters, including the ABC transporter (OpuCA and OpuCC), probable export protein (Lmo2463), and phosphotransferase system component IID (Lmo0781); stress response proteins, including a protein similar to *Bacillus subtilis* general stress protein (Lmo0211) and catalase (Lmo2785); metabolic proteins, including one similar to tagatose-1, 6-diphosphate aldolase (LacD); translational proteins including bacterial protein translation initiation factor IF-3 (InfC); and cellular processing proteins including cell division protein (FtsZ).

**Table 1:** Extracellular membrane vesicle (MV) proteins identified by LC-ESI-MS/MS analysis as regulated by σ^B^ in wild- type *L. monocytogenes*.

**Category**	**Protein Name**	**Description [Reference]**
Transporters	OpuCA	Glycine betaine/carnitine/choline ABC transporter (ATP-binding protein) [6]
	OpuCC	Glycine betaine/carnitine/choline ABC transporter (osmoprotectant-binding) [6]
	Lmo2463	Probable export protein [6]
	Lmo0781	Phosphotransferase system (PTS) component IID [29]
Stress	Lmo0211	Similar to *Bacillus subtilis* general stress protein [29]
	Lmo2785	Catalase [5]
Metabolism	Lmo1694	Epimerase, NAD-dependent family
	Lmo0539 (LacD)	Similar to tagatose-1, 6-diphosphate aldolase [29]
	Lmo1694	Similar to CDP-abequose synthase [29]
	Lmo0722	Similar to pyruvate oxidase [29]
Translation	Lmo1785 (InfC)	Bacterial protein translation initiation factor IF-3 [5]
Cellular processes	Lmo2032 (FtsZ)	Cell division protein [5]
Unknown	Lmo2673	Conserved hypothetical ATP-binding domain [6]
	Lmo0953	Hypothetical protein [29]
	Lmo1257	Hypothetical protein [29]
	Lmo1261	Hypothetical protein [29]
	Lmo0796	Conserved hypothetical protein [29]
	Lmo2673	Conserved hypothetical protein [29]

### Functional classification of the proteins in MVs derived from L. monocytogenes

**Figure 3:**
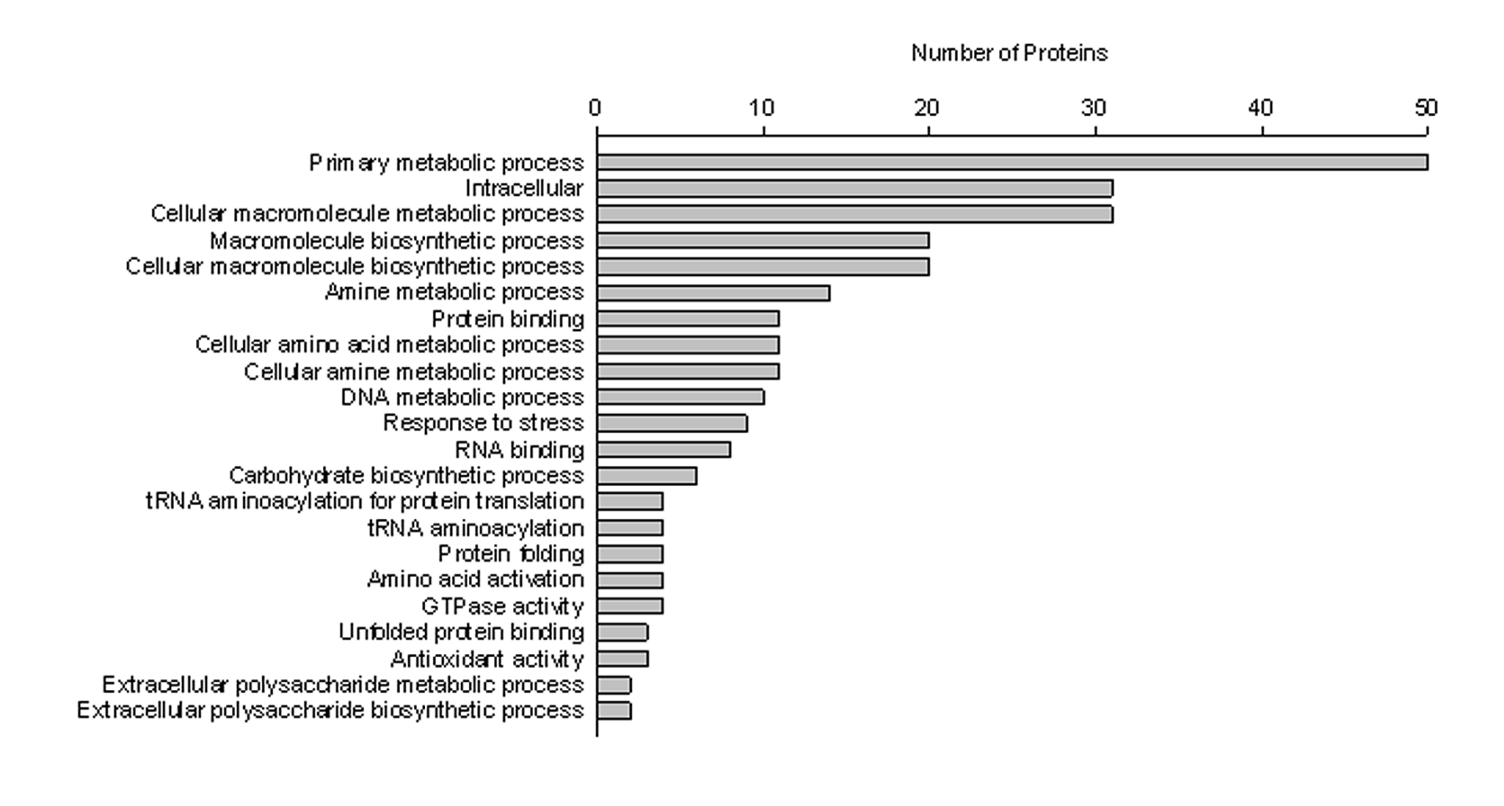
Distribution of significant Gene Ontology (GO) terms from extracellular membrane vesicle (MV) proteins that were categorized only in wild-type *L. monocytogenes.*

A GO enrichment analysis was performed to categorize the functions of the proteins identified in the MVs. A complete list of all GO terms and their assigned functional groups is provided in Tables S4 and S5. A total of 130 MV proteins from the wild-type strain and 89 MV proteins from the ∆*sigB* mutant were commonly categorized in 48 significant GO terms (Table S4). The most significantly enriched GO terms related to molecular functions in both strains included binding; ATP, ribonucleotide, drug and rRNA binding; ligase activity, structural molecule activity and DNA topoisomerase. The most significantly enriched GO terms related to biological processes in the both strains included cellular processes, metabolic processes; cellular protein, macromolecule and organic acid metabolic processes; and translation. Some of the significantly affected cellular components in both strains were the cytoplasm, organelles, cytosolic ribosomes, and the macromolecular complex. Besides the 48 overlapped significant GO terms in the MV proteins derived from both strains, 22 significant GO terms were enriched only in the wild-type *L. monocytogenes* (Table S5). As shown in Figure 3, the most significant affected GO terms related to biological processes were metabolic processes; cellular macromolecules, amines, cellular amino acids, DNA, extracellular polysaccharide metabolic processes; biosynthetic processes; macromolecules, carbohydrate and extracellular polysaccharide biosynthetic processes; stress response, protein folding, tRNA aminoacylation, and amino acid activation. The most significantly enriched GO terms related to molecular functions were binding; unfolded proteins, RNA and protein binding; GTPase activity, and antioxidant activity.

### Identification of virulence factors InlB and LLO in the L. monocytogenes MVs

**Figure 4:**
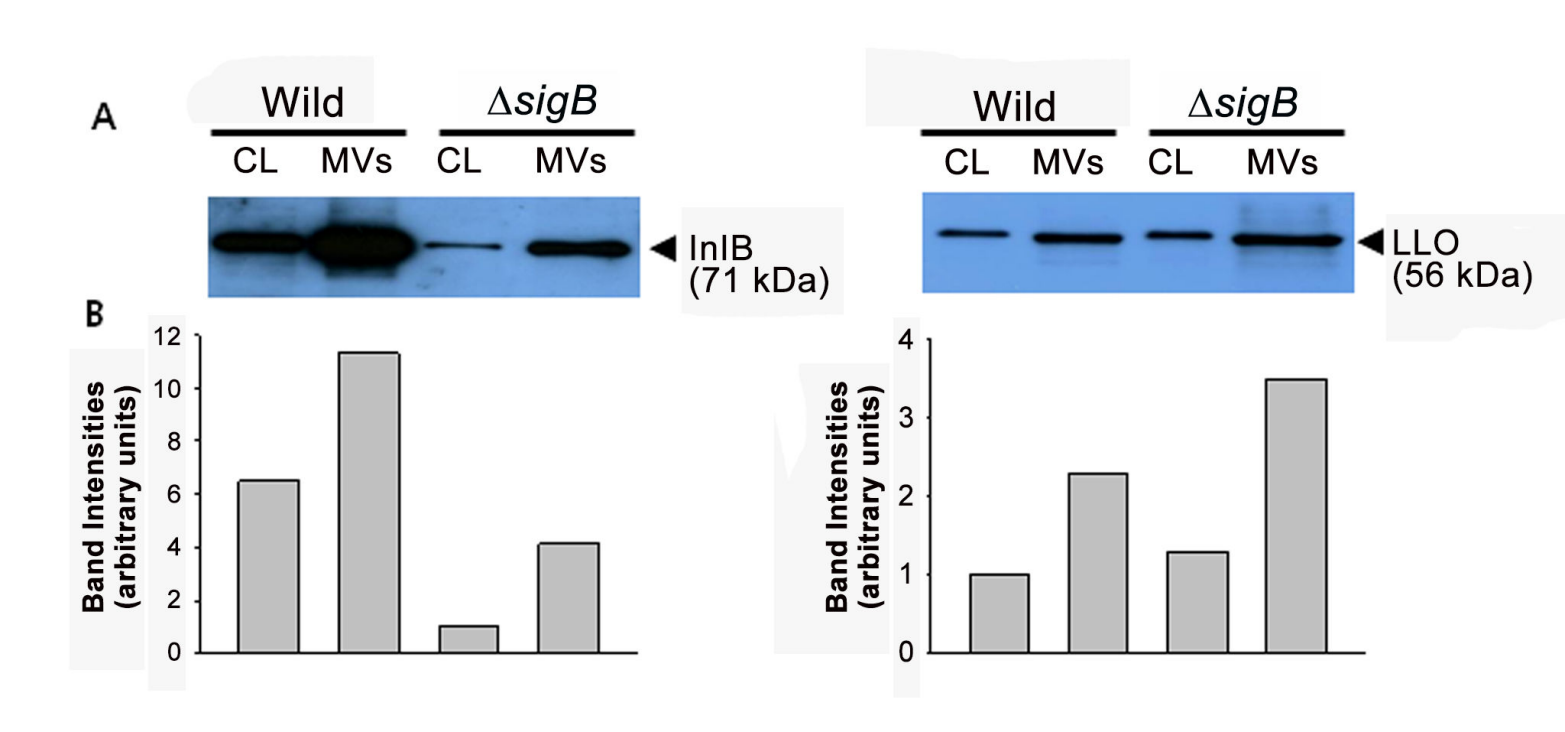
Western blot analysis of Internalin B (InlB) and Listeriolysin O (LLO) in the cell lysate and MVs. (A) Samples were separated on 10% SDS-PAGE and immunoblotted with anti-InlB and anti-LLO antibodies. CL, *L. monocytogenes* cell lysate; MVs, membrane-derived vesicles. (B) Band intensities were measured using image analysis software.

The major virulence factors, InlB and LLO, needed for entry of *L. monocytogenes* into host epithelial cells and for vacuolar lysis, respectively, were identified in both wild-type and ∆*sigB* mutant *L. monocytogenes* MVs. InlB is regulated by both the σ^B^ transcription factor and the positive regulatory factor A (PrfA), whereas LLO is regulated only by a PrfA [30]. Western blot analyses were performed to determine whether InlB and LLO were secreted from bacteria *via* MVs and whether their secretion was affected by σ^B^. Twenty µl of cell lysate (CL) and MVs (1.65 µg for InlB and 20 µg for LLO) from the wild-type strain and ∆*sigB* mutant were separated on 10% SDS-PAGE and immunoblotted with anti-InlB and anti-LLO antibodies. As shown in Figure 4, InlB was 6.5 times more highly expressed in the wile-type cell lysate than in the ∆*sigB* mutant cell lysate, and InlB was almost three times more contained in MVs derived from the wild-type strain than in MVs derived from the ∆*sigB* mutant. However, the LLO level between the wild-type strain and ∆*sigB* mutant was not different in either cell lysates or MVs.

## Discussion

We first demonstrate production of MVs from culture supernatants of food-borne Gram-positive pathogen *L. monocytogenes*. This result supports reports of MV production in other Gram-positive bacteria, such as *S. aureus* [18,19], *Bacillus* spp. [20,21], and *M* *. ulcerans* [22]. The production and release of MVs into the extracellular milieu appears to be conserved among both Gram-negative and Gram-positive bacteria. Interestingly, OMVs or MVs released from pathogenic bacteria contribute to bacterial pathogenesis, as they are involved in the delivery of toxins or virulence factors to eukaryotic cells [11,21,31]. We demonstrated that the general stress transcription factor σ^B^ played a pivotal role in MV production of *L. monocytogenes*. Furthermore, a proteomic analysis demonstrated that MVs derived from the wild-type strain contain important virulence factors needed for host infection. The GO enrichment analysis showed that the functional categories of proteins contained in MVs were significantly different between the wild-type strain and ∆*sigB* mutant. Therefore, our results extend the role of *L. monocytogenes* σ^B^ in the bacterial secretion system through MV production.

Wild-type *L. monocytogenes* produced about nine times more MVs than the ∆*sigB* mutant. Moreover, unlike the wild-type strain-derived MVs, which showed round-shaped nanovesicles, MVs derived from the ∆*sigB* mutant were deformed (Figure 1A and 1B). Similarly, enterotoxigenic *E. coli* produces more OMVs than nonpathogenic *E. coli* [32] and loss of *yfgL*, an encoded lipoprotein involved in the synthesis and/or degradation of peptidoglycans, causes reduced production of OMVs in adherent-invasive *E. coli* [33]. Although these findings were obtained from Gram-negative bacteria, we infer that Gram-positive *L. monocytogenes* σ^B^ may be related to increased production of MVs to promote survival under harsh environments or during infection. In addition, *L. monocytogenes* σ^B^ possibly contributes to monitoring and maintaining cell wall integrity by regulating certain genes [7,9]. In this study, both *L. monocytogenes* were grown in BHI broth until the stationary growth phase and this energy stressed condition may have affected cell envelope function, particularly in the ∆*sigB* mutant, which may have caused the deformity in the MVs. The shapes of the MVs from *L. monocytogenes* were bilayered spherical vesicles, which was the same as MVs released from other Gram-positive bacteria, but the size was more similar to *S. aureus* MVs (20–100 nm in diameter) [18,19] than *B. anthracis* MVs with average diameters of 50–300 nm [21].

We conducted a proteomic analysis with purified MVs derived from *L. monocytogenes* to understand the pathophysiological role of MVs. About 1.5 times more proteins were found in the wild-type strain-derived MVs than in the ∆*sigB* mutant-derived MVs. The major virulence factors InlB and LLO were identified among the commonly identified 84 MV proteins produced from both the wild-type strain and ∆*sigB* mutant (Table S1). InlB is required for adhesion and invasion of *L. monocytogenes* into host cells [34,35], and this protein is co-regulated by both σ^B^ and PrfA, which is directly regulated by σ^B^ [30,36]. The pore-forming toxin LLO is essential for escape of *L. monocytogenes* from a phagosomal compartment into the cytosol and is also required for productive cell to cell spread [30,37,38]. The immunoblotting data showed that InlB was three higher times in wild-type strain-derived MVs than in ∆*sigB* mutant-derived MVs, whereas LLO, which is regulated only by PrfA, was contained in MVs from both strains with similar amounts (Figure 4). These results suggest that MVs from *L. monocytogenes* contain important virulence proteins like other pathogenic bacteria-derived MVs, such as *B. anthracis* MVs [21] and *S. aureus* MVs [18], and σ^B^ also contributes to the secretion of virulence factors contained in MVs.

Among the 46 MV proteins identified only in the wild-type strain, many proteins (39%, 18/46) were identified as regulated by σ^B^. Notably, OpuCA and OpuCC, which are osmolyte transporters, importantly contribute to *L. monocytogenes* survival in the lumen of the small intestine and the duodenum with increased osmotic pressure [3,39,40], or under low temperature conditions [41]. In addition, stress response, metabolism, translation and cellular process-related proteins were identified (Table 1). From these results, we demonstrated that *L. monocytogenes* σ^B^ is involved not only in the containing of virulence proteins but also in the containing of stress-protecting proteins in MVs.

In the GO enrichment analysis using both *L. monocytogenes*-derived MVs, the most significantly enriched GO terms included binding (MF) and metabolic and cellular processes (BF) in both the wild-type strain and ∆*sigB* mutant (Table S4), whereas information storage and processing such as transcription and translation, metabolism, and multi-organism processes are the most enriched GO terms in *S. aureus*-derived MVs [18]. The most significantly affected cellular component (CC) in both *L. monocytogenes* strains was the cytoplasm, which was similar to that observed in the two proteomes of *S. aureus*-derived MVs [18,19]. Besides the commonly categorized 48 significant GO terms in MV proteins produced from both *L. monocytogenes* strains, MV proteins derived from the wild-type strain were categorized into 22 GO terms (Table S5). The GO term for the stress response included nine stress response related proteins, including Kat (catalase), which contributes to growth of *L. monocytogenes* under low temperature [42]; ClpC (endopeptidase Clp ATP-binding chain) and ClpB (ATP-dependent Clp protease), DnaJ (heat-shock protein DnaJ), and DnaK (heat-shock protein DnaK), which are needed for heat shock [43]; UvrA (excinuclease ABC), which is required for acid and bile resistance in *L. monocytogenes* [44] and ReA, which contributes to acid and bile salt stress as well as adhesion and invasion of Caco-2 cells in *L. monocytogenes* [45]. Moreover, two proteins, Lmo1084 (similar to DTDP-L-rhamnose synthetase) and Lmo1081 (similar to glucose-1-phosphate thymidyl transferase) of the extracellular polysaccharide biosynthetic process were categorized into biological processes. Extracellular polysaccharide is an important component of biofilms, which are structured communities of microorganisms enveloped with self-produced biopolymer known as extracellular polymeric substances [46]. OMVs are a definite component of *P. aeruginosa* biofilms [47]. In the GO term analysis, we demonstrated that wild type *L. monocytogenes*-derived MV proteins had important functions for survival under various stressful environmental conditions, adhesion and invasion of intestinal epithelial cells, and serving as biofilm components, but those were not observed in the ∆*sigB* mutant.

In conclusion, we have provided important data about the new protein secretion system of *L. monocytogenes via* MVs. Wild-type strain-derived MVs contained a higher amount of major virulence factor InlB than Δ*sigB* mutant-derived MVs, and these MVs also significantly contained stress response proteins regulated by σ^B^, which play pivotal pathological functions during infection. Our results provide the first observation that transcription factor σ^B^ contributes to the number of MVs produced and the kinds of proteins contained in the MVs. The challenge for future studies is to understand how the MVs specifically contribute to pathogenesis *in vivo*.

## Supporting Information

Figure S1 Growth and σ^B^ activity of wild-type *L. monocytogenes* and Δ*sigB* mutant.
(PPTX)

Table S1 Extracellular membrane vesicle (MV) proteins identified by LC-ESI-MS/MS in both the wild-type and Δ*sigB* mutant *L. monocytogenes*.
(XLS)

Table S2 Extracellular membrane vesicle (MV) proteins identified by LC-ESI-MS/MS in only wild-type *L. monocytogenes*.
(XLS)

Table S3 Extracellular membrane vesicle (MV) proteins identified by LC-ESI-MS/MS in only the Δ*sigB* mutant *L. monocytogenes*.
(XLS)

Table S4 Gene Ontology (GO) terms that were significant (*p* < 0.05) in the extracellular membrane vesicle (MV) proteins derived from both wild-type and Δ*sigB* mutant *L. monocytogenes*.
(XLS)

Table S5 Gene Ontology (GO) terms that were significant (*p* < 0.05) in the extracellular membrane vesicle (MV) proteins derived from only wild-type *L. monocytogenes*.
(XLS)
