# Supplementary material for: Transcription Factor σB Plays an Important Role in the Production of Extracellular Membrane-Derived Vesicles in Listeria monocytogenes
Source: PLoS One. 2013 Aug 20;8(8):e73196. doi: 10.1371/journal.pone.0073196 (PMC3748028; doi:10.1371/journal.pone.0073196)
Supplement: Figure S1 — Growth and σB activity of wild-type L. monocytogenes and ΔsigB mutant. (PPTX) [file pone.0073196.s001.pptx]

## Slide 1
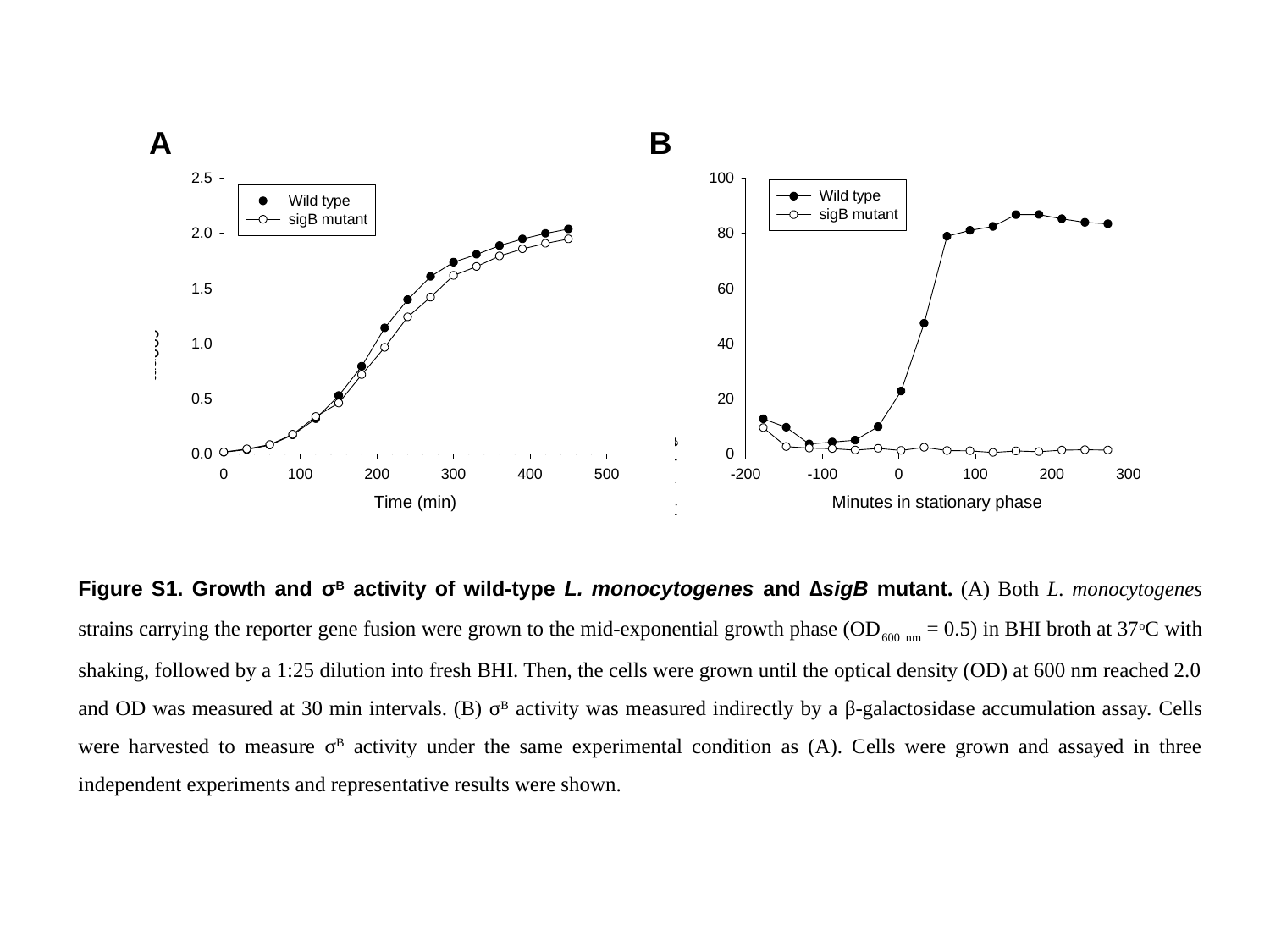

A
B
Figure S1. Growth and σB activity of wild-type L. monocytogenes and ∆sigB mutant. (A) Both L. monocytogenes strains carrying the reporter gene fusion were grown to the mid-exponential growth phase (OD600 nm = 0.5) in BHI broth at 37oC with shaking, followed by a 1:25 dilution into fresh BHI. Then, the cells were grown until the optical density (OD) at 600 nm reached 2.0 and OD was measured at 30 min intervals. (B) σB activity was measured indirectly by a β-galactosidase accumulation assay. Cells were harvested to measure σB activity under the same experimental condition as (A). Cells were grown and assayed in three independent experiments and representative results were shown.
